# The effect of anthropogenic and natural factors on the prevalence of physicochemical parameters of water and bacterial water quality indicators along the river Białka, southern Poland

**DOI:** 10.1007/s11356-018-1212-2

**Published:** 2018-01-30

**Authors:** Anna Bojarczuk, Łukasz Jelonkiewicz, Anna Lenart-Boroń

**Affiliations:** 1 Department of Hydrology, Institute of Geography and Spatial Management, Jagiellonian University in Cracow, Gronostajowa 7, 30-387 Cracow, Poland; 2 Department of Microbiology, University of Agriculture in Cracow, Mickiewicza Ave. 24/28, 30-059 Cracow, Poland

**Keywords:** Bacterial indicators, Physicochemical parameters, River Białka, Water quality

## Abstract

This study was aimed to determine the anthropogenic and natural factors affecting spatial and temporal changes in the physicochemical parameters and bacterial indicators of water quality in the river Białka. The impact of intensive development of the tourist infrastructure on the quality of river water and the potential health threats to tourists was also assessed. Water samples were collected over a period of 2.5 years, once per each month in four sites along the river. Temperature, electrolytic conductivity, pH, and water level were measured onsite; flow rate data were acquired from the Institute of Meteorology and Water Management; chemical analyses allowed to determine the amount of fourteen ions, while microbiological indicators included total and thermotolerant coliforms, total and thermotolerant *Escherichia coli*, and mesophilic and psychrophilic bacteria. The combination of hydrological, hydrochemical, and microbiological methods generated large amount of data, which were processed by multivariate statistical analysis. A downstream cumulative effect was observed in the contamination of the river water. Fecal coliforms and *E. coli* were detected in all sites, suggesting the source of fecal contamination even in the protected areas. Intensive development of a ski resort and the related infrastructure, together with the need to accommodate numerous tourists in the examined region, has an evident environmental impact. The resulting deterioration of water quality poses health risks to tourists, as water from the Białka river is used for a variety of purposes, including as a raw drinking water or for artificial snowing of ski slopes. The seasonal changes in the physicochemical parameters mainly result from varying natural factors that shape the water quality in the studied region. The differences in the number of analyzed microorganisms result from seasonal variation in touristic activity and are affected mostly by point sources of sewage inflow.

## Introduction

Water resources in Poland are very diverse—both spatially and temporally, but in general, Poland in comparison with other European countries is classified as having very limited water resources (Orlińska-Woźniak et al. 2013). One of the most important problems of water management in Poland is not the lack of water in general, but the lack of water in the right place and of adequate quality. Water quality is among the most important factors affecting health and safety of its users and the suitability for its utilization in various aspects (Lenart-Boroń et al. 2017b). The insufficient quality results from an increasing pollution of surface water, mostly in rivers, resulting from discharge of municipal and industrial sewage and surface runoff carrying large amounts of fertilizers from agricultural fields (Pawełek 2015).

Mountain areas are among the most important and most valuable areas in the world. They are characterized by a huge biodiversity, but they are also very sensitive to natural and anthropogenic changes. Thirty-two percent of protected areas in the world are located in the mountains. Almost half of the world’s population is dependent on mountain areas. The mountains are called “water towers,” because they supply nearly half of the world’s population with water (Viviroli et al. 2003; Korner and Ohsawa 2005). The continuous development of tourism in mountain areas, especially winter tourism and the development of ski resorts, affects the natural environment (Tsuyuzaki 1994; Kangas et al. 2009). It is often emphasized that one of the most important aspects is the impact of tourism on resources and quality of water. Ski resorts, in addition to water intake for artificial snowing, also deteriorate the quality of water in rivers and streams due to the contamination with nutrients, bacteria, and other microorganisms by treated and untreated human waste (Pickering et al. 2003; Kangas et al. 2009; Wemple et al. 2007). Also in connection with the progressive global warming, the demand for water for snowing of ski slopes increases. The largest water intake in ski resorts occurs at the beginning (for the so-called base snowmaking) and at the end of the winter period (to extend the skiing season) (Vanham 2011).

In most cases, the quality of water in catchments is subject to both temporal and spatial changes and—depending on the specificity of the catchment or region—these changes are the result of various combinations of natural and/or anthropogenic factors (Lenart-Boroń et al. 2016a, 2017b; Ouattara et al. 2011; Huang et al. 2014). Natural factors include geological structure, seasonal differences in runoff volumes, weather conditions, water levels and flows, land cover, and the growing cycle (Bartram and Ballance 1996). Among anthropogenic factors, one can mention land use, which in turn affects the type, number and location of point, and non-point sources of pollution (USEPA 2015). In the case of the studied region, i.e., the Bukowina Tatrzańska municipality and the Tatra poviat, the water resources are mainly affected by the specificity of the area, where tourism has recently undergone very intensive development (Krąż 2012a). According to Janczy (2015), the Tatra poviat has more than 23,000 accommodation places and the annual number of guests exceeds 2.2 million. This generates numerous point sources of pollution, such as discharge of sewage from an ineffectively operating municipal sewage treatment plant and plenty of small illegal discharge sites from households (Lenart-Boroń et al. 2016a). Also the quantity of water is affected by the expansion of winter tourism, as it generates increased consumption of water resources. This is due to the large numbers of tourists visiting the region during the so-called “high ski season,” resulting in increased use of water for living purposes, but also due to the fact that in order to improve the conditions on the ski slopes, the ski stations use snow cannons for artificial snowing. The standard snow cannons may take more than 1400 m^3^/h of water, and there are eight ski stations in the Bukowina Tatrzańska municipality that use such ways of extending ski season (Krąż 2012b). The above described problems are currently observed worldwide, as mountain hiking and winter tourism have become one of the most important economic sectors in various mountain areas, in different regions of the world (Rixen et al. 2003). This is because the tourism and outdoor recreation are among the forms of intensive land use, with ski resort infrastructure being one of the main factors causing environmental degradation in various mountain regions of the world (Kangas et al. 2009).

Having regard to the above listed aspects, identification of phenomena affecting the quality of water in the catchment and understanding of the prevailing factors determining water quality is not only important for the sake of natural environment, but it is also a difficult task. Applying a range of tools might improve our understanding of mechanisms affecting the quality of water in ecosystems that are sensitive to various factors, including both natural and man-induced disturbances, such as the mountain rivers (Esposito et al. 2016). The quality of water in general is the combination of the number of microorganisms, concentrations of chemical compounds, and physical properties; therefore, interdisciplinary studies provide the best effects in this respect. Combining hydrochemical testing with microbiological analyses of water can be a useful tool enabling multivariate analysis of obtained results, and it has proved useful in a number of studies on aquatic ecosystems, including high-alpine spring waters in Italy (Esposito et al. 2016), groundwater of the Mamora basin in Morocco (Kabbour and Zouhri 2005), or groundwater of the Merdja plain in Algeria (Fehdi et al. 2016). Harclerode et al. (2013) successfully combined a geographical approach with microbiological and chemical analyses of water to track the point and non-point sources of contamination with *E. coli* in Texas. Bhandari et al. (2017), on the other hand, conducted an analysis taking into consideration a number of water quality-affecting factors, including the amount of heavy metals, *E. coli* concentration and land use in a watershed of Brays Bayou (Texas) to track the changes in the land use and water pollution of an economically important river. Also in Poland, a combination of methods, i.e., physical, microbiological, hydrological, and meteorological allowed for obtaining a large set of data that were subjected to multivariate statistical analysis. This enabled tracking the factors affecting spatiotemporal changes in a number of water quality indicators, to assess the gradient of pollution along a river and to determine the cumulative effect of urban pollution (Glińska-Lewczuk et al. 2016).

In this study, a combination of hydrological, hydrochemical, and microbiological data was used to identify factors determining the spatial and temporal changes in the physicochemical parameters and the prevalence of bacterial indicators of water quality in a mountain ski resort based on the example of the river Białka, southern Poland. Another aim was the attempt to assess the impact of the development of a mountain resort on the quality of water in the Białka river and the possible health risks for tourists.

## Material and methods

### Study site

The study was conducted in the catchment of the Białka river within the Tatra Mountains and Podhale in the Bukowina Tatrzańska municipality. This area is among the most attractive tourist destinations in Poland. The studied river flows from the southern part of the Tatras—the highest mountain range in Poland. Southern part of the river catchment is the protected area (Tatra National Park and Natura 2000) and has undergone only small anthropogenic transformation (more than 60% of the area is covered by forests). Ski tourism is a very important branch of business in the studied area. Due to highly developed ski infrastructure and tourist accommodation, the considered region is characterized not only by a high-water demand for municipal purposes and for artificial snowing of ski slopes, but also by large amounts of produced wastewater. Unfortunately, only 49.8% of the Bukowina Tatrzańska municipality citizens use the local sewage treatment plant (CSO 2013). This results in a number of illegal sewage discharge sites to the nearby river, Białka, and its tributaries. In addition to that, the number of the municipality residents increases several times in the winter touristic season, resulting in the sewage treatment plant overload and decreased effectiveness of its operation (Lenart-Boroń et al. 2016a).

### Field methods

**Fig. 1:**
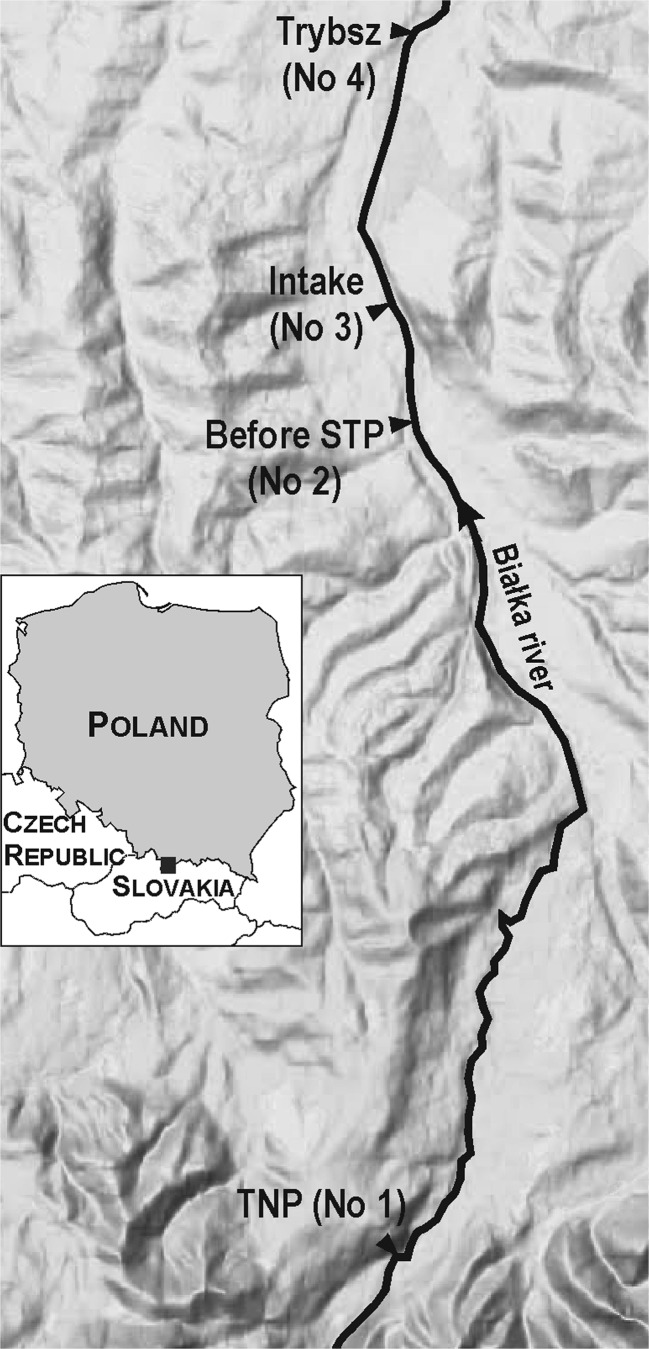
Study area and the location of the sampling sites

The samples were collected from January 2013 to June 2015 in four sites along the river Białka. The location of the sampling sites is shown in Fig. 1. Site no. 1 (TNP) is situated on the border of the Tatra National Park. Site no. 2 (Before STP) is situated a few hundred meters upstream of the sewage discharge from the sewage treatment plant of the Bukowina Tatrzańska municipality. Site no. 3 (Intake) is the place of water intake for artificial snowing of one of the largest ski stations in Poland (Kotelnica Białczańska) and is located downstream of the sewage discharge from the mentioned treatment plant. Site no. 4 (Trybsz) is situated at the border of the Bukowina Tatrzańska municipality.

Water temperature (T), electrolytic conductivity (EC_25 °C_), pH, and water level (H) were measured onsite (Lenart-Boroń et al. 2016c). Water samples were collected each month to 1000 ml sterile polypropylene bottles for the assessment of chemical composition and into 500 ml polyethylene bottles for microbiological analyses. Data concerning flow rate in the Białka river were acquired from the Institute of Meteorology and Water Management—National Research Institute.

### Laboratory methods

Chemical composition of water was determined at the laboratory of the Institute of Geography and Spatial Management of the Jagiellonian University in Cracow. After filtration of water with 0.45 μm a syringe filter, the chemical composition was determined with ion chromatography using a combined system of two ion chromatographs DIONEX ICS-2000 and an autosampler AS-4 (Lenart-Boroń et al. 2016c). Water mineralization (TDS) was calculated as a sum of the detected ions. Statistical analysis was based on the parameters: T, EC, pH, TDS, and nitrogen and phosphorus compounds (NH_4_^+^, NO_3_^−^, NO_2_^−^, PO_4_^3−^).

In microbiological analyses, bacterial indicators of water quality were enumerated with membrane filtration (total and thermotolerant coliforms and *Escherichia coli*) and serial dilution methods (mesophilic and psychrophilic bacteria), as described by Lenart-Boroń et al. 2016c.

### Statistical analysis

Principal Component Analysis (PCA) was employed to determine the relationship between the microbiological indicators and the chemical quality parameters, and amount of water, as well as to explain the natural and anthropogenic processes that affect these parameters. PCA was calculated for each of the study sites based on the following variables: the numbers of total and fecal (thermotolerant) coliforms, total and fecal *E. coli*, and mesophilic and psychrophilic bacteria, as well as T, pH, TDS, the concentration of NH_4_^+^, NO_3_^−^, NO_2_^−^, PO_4_^3−^ions, and a flow rate (Q). The number of the most important factors was determined based on the Kaiser criterion and explained by the factor of variance above 10%. Analysis of variance (ANOVA) and a post hoc Scheffe test for *p* = 0.95 were used to determine the significance of differences in the examined parameters between the study sites and seasons of the year. Spearman’s rank correlation coefficients were calculated in order to determine whether there are relationships between microbiological indicators, physicochemical parameters of water, and a flow rate. Statistical calculations were conducted in STATISTICA 12.5 software (StatSoft, USA).

## Results

### Basic characteristics of water parameters

Table 1 shows basic statistical characteristics and coefficient of variation (CV) for microbiological and physicochemical parameters of water and flow rate in the study sites. Total coliforms are the predominant group of bacteria in water, and *E. coli* (both total and thermotolerant groups) are the least numerous. Water pH is mostly alkaline and TDS—low (max. 251.3 mg/L). Among the nitrogen compounds, nitrates have the highest concentrations and nitrites—the lowest. The concentrations of PO_4_^3−^ions in the studied sites are very low and do not exceed 0.5 mg/L. Coefficients of variability for microbiological indicators, as well as nitrogen and phosphorus compounds, are very high, which indicates large fluctuations in values of these characteristics during the study period. An increase in the mean values of microbiological and physicochemical characteristics of water along the course of the river can be noticed.

**Table 1:** Basic statistical characteristics of microbiological indicators, physicochemical parameters, and water flow rates in the studied sites. Number of collected samples throughout the study is *n* = 32

Site		TNP (No 1)		Before STP (No 2)		Intake (No 3)		Trybsz (No 4)	
Feature	Unit	Mean (Min–Max)	CV (%)	Mean (Min–Max)	CV (%)	Mean (Min–Max)	CV (%)	Mean (Min–Max)	CV (%)
Q	m^3^/s	1.29 (0.48–17.50)	123	3.17 (1.46–14.43)	70	3.34 (1.54–15.19)	70	5.21 (1.51–29.60)	87
Total coliforms	CFU/100 ml	1 (0–31,000)	559	243 (0–95,000)	309	1060 (0–78,100)	241	820 (12–54,300)	214
Total *E. coli*		0 (0–50)	341	0 (0–80,000)	512	36 (0–20,000)	241	40 (0–39,100)	240
Fecal coliforms		0 (0–39)	340	21 (0–22,000)	250	6 (0–30,100)	243	620 (0–37,200)	205
Fecal *E. coli*		0 (0–39)	420	0 (0–7000)	280	0 (0–25,390)	279	30 (0–24,000)	259
Mesophilic bacteria	CFU/ml	15 (0–47,000)	443	125 (0–3100)	160	530 (10–14,510)	183	398 (15–27,500)	239
Psychrophilic bacteria		73 (0–13,800)	308	378 (0–24,600)	252	925 (0–42,600)	218	1180 (19–312,000)	449
T	°C	4.9 (3.1–11.0)	42	5.1 (0.0–14.7)	76	4.9 (0.1–15.0)	78	5.3 (0.0–16.0)	82
pH	pH	7.76 (6.81–8.40)	5	8.00 (6.94–8.30)	4	7.99 (7.13–8.46)	4	7.99 (7.10–9.19)	5
EC	μS/cm	123.5 (58.2–290.6)	44	323.3 (126.6–300.0)	21	220.2 (133.7–306.0)	22	233.4 (142.3–322.7)	21
TDS	mg/L	91.3 (42.2–219.8)	45	183.6 (96.1–235.6)	20	178.3 (100.9–241.6)	20	187.7 (108.3–251.3)	19
NH_4_^+^		0.0068 (0.0001–0.6383)	258	0.0135 (0.0001–0.0715)	94	0.0402 (0.0001–0.4216)	131	0.0389 (0.0001–0.5855)	141
NO_3_^−^		2.077 (0.001–4.921)	36	2.526 (1.512–5.488)	37	2.529 (0.001–5.014)	40	2.927 (1.284–5.566)	38
NO_2_^−^		0.0008 (0.0008–0.1076)	288	0.0008 (0.0008–0.5513)	492	0.0008 (0.0008–0.3188)	322	0.0008 (0.0008–0.2777)	191
PO_4_^3−^		0.0033 (0.0013–0.4321)	280	0.0033 (0.0007–0.1458)	251	0.0033 (0.0022–0.4904)	193	0.0033 (0.0026–0.4904)	201

### Spatial heterogeneity

The results of Spearman’s correlation analysis are presented in Table 2. The majority of statistically significant relationships between the numbers of microbiological indicators of water quality, physicochemical parameters, and flow rate were found at the site TNP, while the site Before STP has the smallest number of such relationships. In the sites Before STP and Trybsz, there are generally no statistically significant relationships between microbiological and physicochemical characteristics of water. The flow rate is usually negatively correlated with physicochemical parameters of water. The only exception is the positive relationship between Q and water temperature. The impact of the flow rate on the number of microorganisms in water is visible in the TNP (significant positive relationship with total *E. coli* and fecal coliforms) and Before STP (significant positive relationship with fecal coliforms and mesophilic bacteria).

**Table 2:** Spearman rank correlation coefficients. Bolded values are statistically significant

Site	Feature	Q	Total coliforms	Total *E. coli*	Fecal coliforms	Mes. bact..	Psychr bact.	T	pH	TDS	NH_4_^+^	NO_3_^−^	NO_2_^−^	PO_4_^3−^
TNP (No 1)	Q	1.00	0.18	*0.38*	*0.43*	− 0.22	0.03	*0.60*	*− 0.44*	*− 0.62*	*− 0.41*	*− 0.70*	− 0.14	− 0.24
	Total coliforms		1.00	*0.63*	*0.50*	0.26	0.30	0.35	− 0.27	− 0.32	0.05	− 0.22	0.24	0.06
	Total *E. coli*			1.00	*0.49*	0.12	0.03	*0.42*	*− 0.55*	*− 0.45*	− 0.02	*− 0.36*	0.03	− 0.11
	Fecal coliforms				1.00	0.14	0.16	0.20	*− 0.43*	− 0.30	− 0.26	*− 0.36*	0.17	− 0.09
	Mesophilic bacteria					1.00	0.27	0.01	−0.12	0.13	−0.11	0.16	0.01	0.09
	Psychrophilic bacteria						1.00	0.13	0.22	0.09	0.18	0.24	*0.36*	*0.43*
	T							1.00	*− 0.42*	*− 0.64*	*− 0.45*	*− 0.68*	− 0.21	− 0.27
	pH								1.00	*0.65*	*0.53*	*0.48*	0.19	*0.52*
	TDS									1.00	*0.37*	*0.80*	0.08	*0.58*
	NH_4_^+^										1.00	*0.47*	*0.56*	*0.39*
	NO_3_^−^											1.00	0.28	*0.50*
	NO_2_^−^												1.00	0.13
	PO_4_^3−^													1.00
Before STP (No 2)	Q	1.00	0.08	0.15	*0.39*	− 0.02	*0.36*	*0.67*	− 0.12	*− 0.54*	− 0.13	*− 0.42*	0.07	− 0.20
	Total coliforms		1.00	*0.55*	*0.55*	0.09	0.13	− 0.06	0.05	0.26	0.29	0.28	− 0.19	− 0.24
	Total *E. coli*			1.00	*0.52*	0.32	*0.43*	0.08	0.08	0.18	0.05	0.15	− 0.06	− 0.21
	Fecal coliforms				1.00	0.11	*0.37*	0.18	0.00	0.02	0.12	0.03	0.00	− 0.13
	Mesophilic bacteria					1.00	*0.51*	− 0.02	− 0.34	− 0.12	− 0.21	0.11	0.19	0.10
	Psychrophilic bacteria						1.00	0.33	− 0.07	− 0.08	0.01	− 0.13	− 0.07	− 0.06
	T							1.00	0.07	*− 0.47*	− 0.24	*− 0.67*	− 0.05	− 0.18
	pH								1.00	0.35	0.05	− 0.20	*− 0.43*	− 0.15
	TDS									1.00	*0.38*	*0.66*	− 0.17	0.17
	NH_4_^+^										1.00	0.17	0.08	0.04
	NO_3_^−^											1.00	0.04	0.33
	NO_2_^−^												1.00	− 0.03
	PO_4_^3−^													1.00
Intake (No 3)	Q	1.00	0.25	0.20	0.07	− 0.27	0.21	*0.68*	− 0.09	*− 0.65*	− 0.12	*− 0.40*	− 0.07	− 0.28
	Total coliforms		1.00	*0.63*	*0.66*	0.28	*0.36*	0.14	*− 0.42*	− 0.31	0.15	0.12	0.15	0.24
	Total *E. coli*			1.00	0.72	0.28	0.24	0.11	*− 0.42*	− 0.27	0.01	0.10	0.07	*0.35*
	Fecal coliforms				1.00	*0.40*	*0.42*	− 0.04	− 0.31	− 0.09	0.04	*0.40*	0.11	*0.51*
	Mesophilic bacteria					1.00	*0.38*	− 0.26	− 0.20	0.11	− 0.05	*0.58*	0.29	0.32
	Psychrophilic bacteria						1.00	0.00	− 0.07	− 0.07	0.25	0.33	0.28	0.29
	T							1.00	− 0.07	*− 0.73*	− 0.30	*− 0.55*	− 0.10	*− 0.44*
	pH								1.00	*0.41*	*0.36*	0.10	0.07	− 0.09
	TDS									1.00	*0.43*	*0.50*	− 0.19	0.31
	NH_4_^+^										1.00	0.27	0.24	0.12
	NO_3_^−^											1.00	− 0.02	*0.57*
	NO_2_^−^												1.00	− 0.03
	PO_4_^3−^													1.00
Trybsz (No 4)	Q	1.00	0.28	0.19	0.23	0.27	*0.38*	*0.60*	− 0.15	*− 0.67*	− 0.28	*− 0.43*	0.09	*− 0.51*
	Total coliforms		1.00	*0.73*	*0.86*	0.04	*0.57*	− 0.03	− 0.11	− 0.11	0.22	0.03	0.12	0.07
	Total *E. coli*			1.00	*0.66*	0.21	*0.66*	0.04	− 0.26	− 0.05	0.07	− 0.08	0.19	0.15
	Fecal coliforms				1.00	− 0.04	*0.51*	− 0.01	− 0.10	− 0.16	0.19	− 0.07	0.08	0.07
	Mesophilic bacteria					1.00	*0.49*	0.23	*− 0.40*	− 0.31	− 0.21	− 0.15	0.15	− 0.08
	Psychrophilic bacteria						1.00	0.21	− 0.17	− 0.20	− 0.03	− 0.07	0.31	− 0.07
	T							1.00	− 0.02	*− 0.68*	*− 0.60*	*− 0.63*	− 0.11	*− 0.56*
	pH								1.00	0.32	*0.38*	− 0.08	0.12	− 0.16
	TDS									1.00	*0.67*	*0.55*	0.20	*0.49*
	NH_4_^+^										1.00	*0.37*	*0.52*	*0.35*
	NO_3_^−^											1.00	0.10	*0.46*
	NO_2_^−^												1.00	− 0.05
	PO_4_^3−^													1.00

**Fig. 2:**
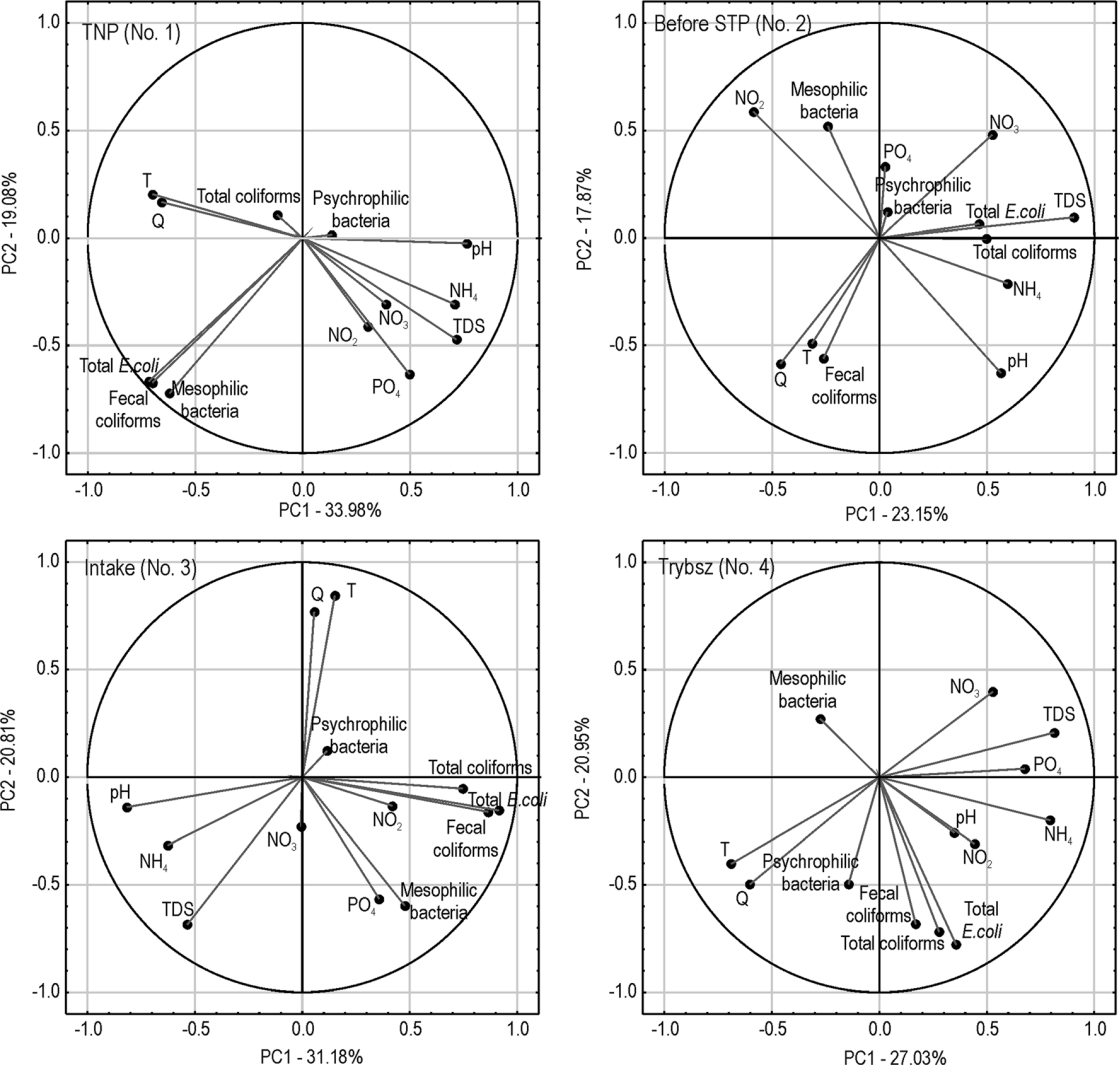
Results of Principal Component Analysis of microbiological indicators of water quality, physicochemical parameters, and water flow for the analyzed sites

Principal Component Analysis showed that the first two factors explain in total from 41.0% (Before STP) to 53.1% (TNP) of variation. The PCA results obtained for the site TNP showed that factor 1 explains 34% and factor 2—19.1% of variation (Fig. 2.). Factor 1 indicates a negative relationship between Q, T, total *E.coli*, fecal coliforms, mesophilic bacteria and pH, TDS, and PO_4_^3−^. In this factor, the higher the flow and water temperature, the more bacteria, but lower mineralization and concentrations of NH_4_^+^ and PO_4_^3−^. On the other hand, factor 2 shows a positive correlation between the number of bacteria (total *E. coli*, fecal coliforms, mesophilic bacteria) and TDS, NO_2_^−^, and PO_4_^3−^. Thus, the more bacteria, the higher concentrations of NO_2_^−^, PO_4_^3−^, and TDS.

In the site Before STP, factor 1 explains 23.1% of variance and factor 2—17.9%. This factor represents a positive relationship between total coliforms, total *E. coli* and pH, TDS, NH_4_^+^, and NO_3_^−^, i.e., the more coliforms and *E. coli*, the higher pH, water mineralization, and higher concentrations of ammonium and nitrates in water. Factor 2 shows a negative relationship between fecal coliforms, Q, pH, T and mesophilic bacteria, NO_3_^−^, and NO_2_^−^. In this relationship, the more fecal coliforms, higher flow, pH and water temperature, the lower concentrations of NO_3_^−^ and NO_2_^−^, and less mesophilic bacteria.

At the Intake factor 1 explains 31.2% of variance and factor 2—20.8%. In factor 1, there is a negative relation between the microbiological indicators of water quality and NO_2_^−^ with pH, TDS, and NH_4_^+^. Thus, the more bacteria and nitrites in water, the lower pH, TDS, and NH_4_^+^. Factor 2 also shows a negative relation between Q, T and mesophilic bacteria, TDS, and PO_4_^3−^. It shows that the higher water flow and temperature, the less mesophilic bacteria, smaller TDS, and lower concentrations of PO_4_^3−^.

In the site, Trybsz factor 1 explains 27% while factor 2–21% of variance. In factor 1, we can observe a negative correlation between Q, T and TDS, and the concentration of nitrogen and phosphorus compounds. This means that the lower flow and temperature of water, the higher concentrations of nutrients. Factor 2 represents a positive correlation between microbiological indicators of water quality and flow and temperature, i.e., the higher flow, the higher water temperature and more bacteria (total and fecal coliforms, total *E. coli* and psychrophilic bacteria) in water.

Analysis of variance (ANOVA) showed that in terms of water temperature and the number of microorganisms, the differences between individual sites are not statistically significant. Only in the case of fecal coliforms, there are significant differences between the sites TNP and Trybsz. The site TNP differs significantly from the remaining sampling sites in the case of Q, pH, EC, and TDS values. Waters in the TNP differ significantly from the site Trybsz in the concentrations of NH_4_^+^ and NO_3_^−^. On the other hand, the site Before STP differs significantly from Trybsz in the concentrations of NO_2_^−^ and PO_4_^3−^. Waters in the TNP are characterized by significantly lower values of Q, pH, EC, and TDS from the other sampling sites. In the case of the concentrations of nitrogen compounds (NH_4_^+^ and NO_3_^−^)—they are significantly lower in the TNP than in Trybsz.

### Seasonal variability

There are no significant differences in the number of microorganisms, water pH, and the concentration of NO_2_^−^ between seasons of the year. In the case of Q, EC, TDS, NH_4_^+^, NO_3_^−^—these values differ significantly in winter from the remaining seasons. Water temperature in each season varies significantly and in the case of PO_4_^3−^ concentration—there are significant differences between spring and winter. ANOVA showed that the values of physicochemical characteristics during winter are significantly higher than in the other seasons, while the water flow rate—significantly lower.

**Fig. 3:**
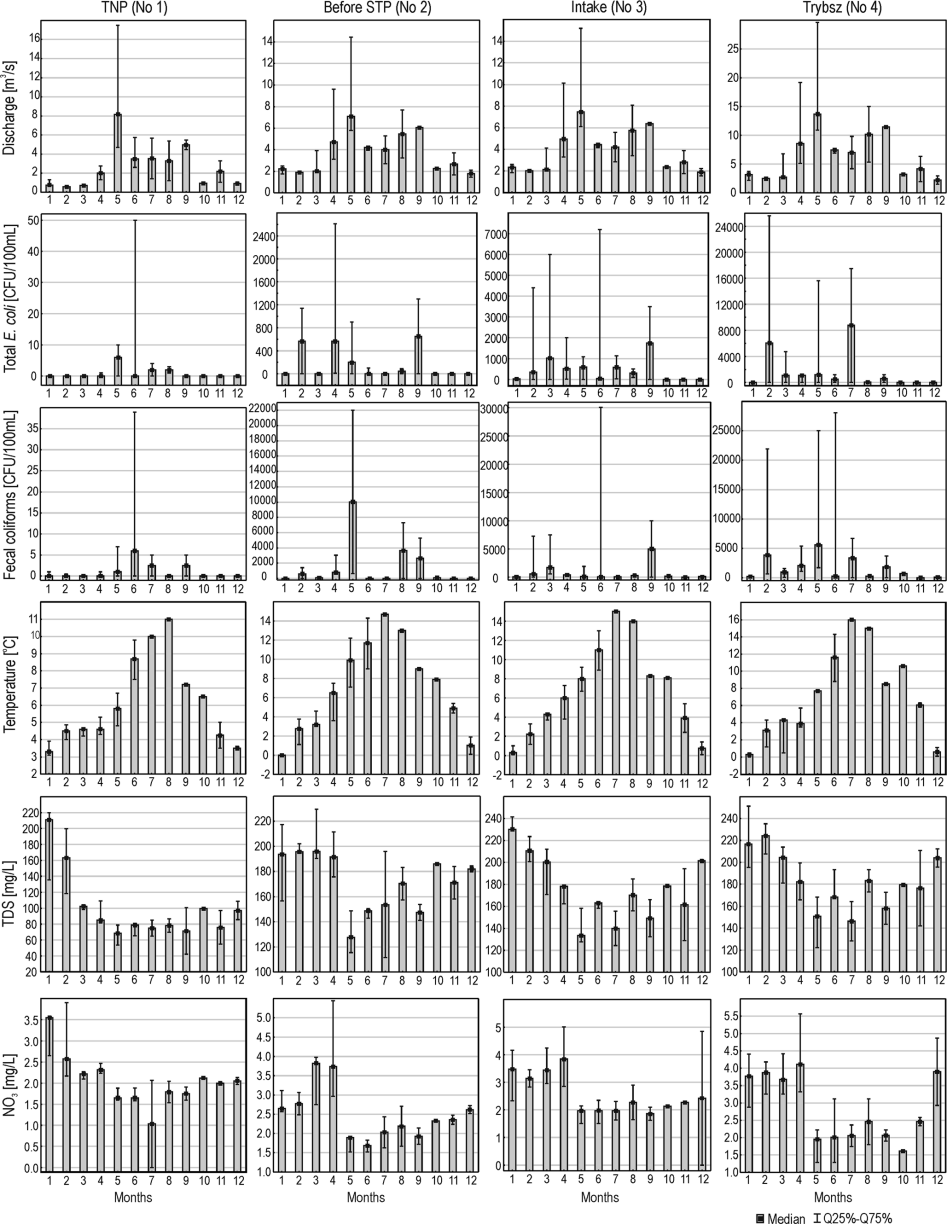
Variation in the number of microorganisms, physicochemical characteristics of water, and flow rate during the year in the analyzed sampling sites

Based on Fig. 3, it can be stated that there is a clear seasonal variability of microbiological indicators of water quality, physicochemical characteristics, and water flow rate. The lowest flow rates occur in winter and autumn, the highest—in spring, and this observation is clear in the case of all sampling sites. The variability in the number of *E. coli* in water corresponds with the flow rate and in the months when the flow rate is higher, also the number of *E. coli* increases. However, high levels of bacteria in water also occur in winter. It should be noted that the number of *E. coli* is very variable in particular months, from year to year, indicated by the large interquartile range. Similarly, as in the case of total *E. coli*, increased number of fecal coliforms can be observed in months with higher flow rates in the river Białka. Only in the sampling sites Intake (No.3) and Trybsz (No.4), greater number of fecal coliforms was observed also in the winter months (February, March). Interquartile range indicates that the number of fecal coliforms in water can differ significantly between individual years. Water temperature is characterized by a very clear seasonality, and it refers to the climatic conditions of the study area. Also TDS refers to the flow rate, but the higher the flow, the lower the TDS values. High values of TDS in the winter months and the lowest during the spring snowmelt period are clearly visible. On the other hand, in most sampling sites, the concentrations of NO_3_^−^ are the highest in the beginning of snowmelt, then drop sharply to remain at low levels during the growing season (spring, summer, and early autumn).

**Fig. 4:**
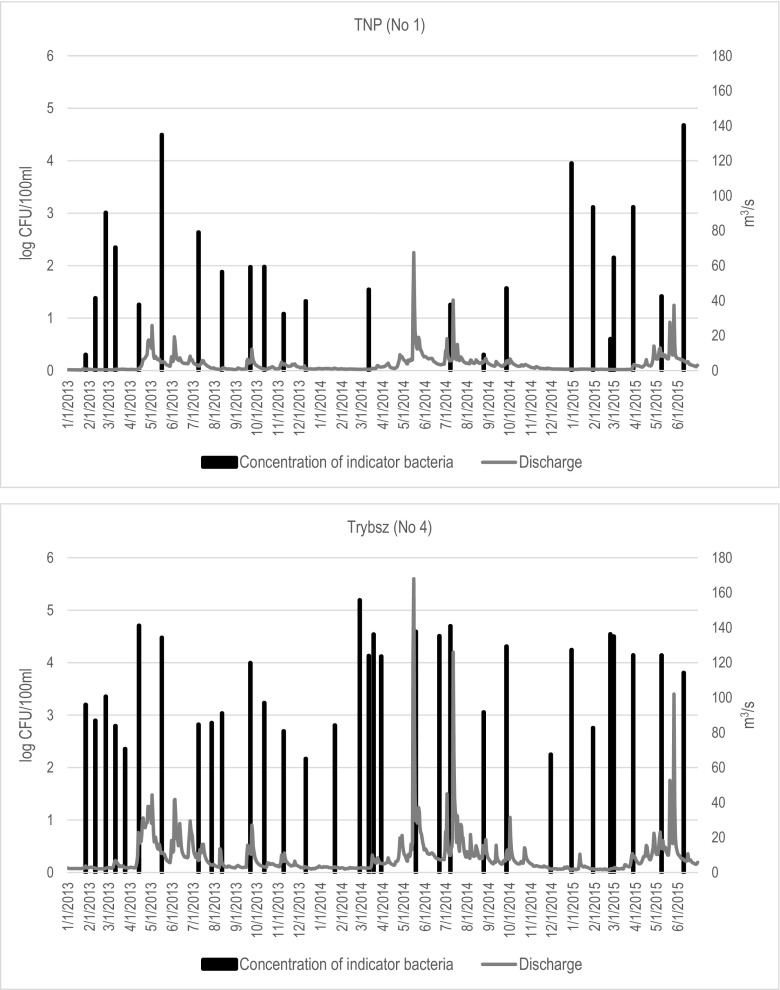
Sum of bacterial water quality indicators and flow rate in the study period in the sampling sites TNP (No. 1) and Trybsz (No. 4)

Fig. 4 shows that in the TNP (No 1), the total number of bacterial indicators in the study period did not change significantly and did not exceed 1000 CFU/100 ml. Their sudden increase was recorded only three times. It is difficult to conclude whether the flow rate affects the number of bacteria, but their highest values occur during higher flow rates. On the other hand, in Trybsz (No 4), the total number of bacteria changes clearly over the study period. Their highest levels were recorded in 2014. It can be noticed that the number of bacteria in waters increases during the flood waves.

## Discussion

Increasing numbers of bacteria and physicochemical characteristics of water can be observed along the course of the river Białka. However, in terms of microbiological indicators, these changes are not significant, unlike physicochemical characteristics. Increasing values of microorganisms and physicochemical parameters along the course of the river is a common observation (Sinclair et al. 2009; Lenart-Boroń et al. 2017b), but in terms of bacterial indicators, these changes are not always statistically significant (Stocker et al. 2016). Such increase in the concentration of pollutants results from the fact that fecal contamination in rivers has a downstream cumulative effect (Ponce-Terashima et al. 2014). Even though total coliforms were the predominant group of microbial indicators of water quality, the presence of thermotolerant *E. coli* was also detected, even at the site located in the Tatra National Park (No. 1). Also An and Breidenbach (2005) detected total coliforms in all and *E. coli*—in 78% of spring water samples collected in recreational mountain areas in South Korea, suggesting malfunctioning septic systems and wildlife population to be the source of contamination. The presence of these bacteria may pose a health risk to the tourists who sometimes use this water as raw drinking water during hiking.

Principal Component Analysis (PCA) allowed to distinguish two main factors, affecting the variability of microbiological indicators of water quality and physicochemical parameters, for each of the sampling site. In the TNP (No. 1), the first factor is associated with dilution of ions and at the same time leaching bacteria from soils. In factor 2, the positive correlation between the number of total *E. coli*, fecal coliforms, and mesophilic bacteria with the concentration of PO_4_^3−^ may indicate the effect of anthropogenic pressure in the form of inflow of human and animal feces. This could be due to the numerous hiking trails in the close proximity and horse carriages transporting tourists to the Morskie Oko—the largest lake in the Tatra Mountains, which is extremely popular destination of tourist hikes. Similar conclusions were presented by An and Breidenbach (2005) with respect to the contamination of spring waters with total coliforms and *E. coli*. On the other hand, Lenart-Boroń et al. (2016a) demonstrated that both factors affecting the variability of the examined parameters in the same sampling site on the river Białka are “natural,” i.e., associated with surface runoff and supply of ions and bacteria from snowmelt water.

At the sampling site No. 2—Before STP, the observed relationships are not obvious and may indicate—similarly as demonstrated in the study of Lenart-Boroń et al. (2016a, c)—the mixed effect of anthropogenic pressure and natural factors. The inflow of sewage affects the presence of microorganisms—both by supplying some of them and by decreasing the number of others. Natural factors affecting the variation of the examined parameters include changing weather conditions, growing cycle, snowmelt, soil leaching, and surface runoff (Lenart-Boroń et al. 2016a, c).

At the Intake (No. 3), the first factor may be associated with anthropogenic pressure in the form of inflow of fecal contamination either from the nearby sewage treatment plant or from illegal discharge of sewage from households not connected to the sewerage system and leaching bacteria from soils in periods with increased flows, i.e., with snowmelt, heavy rainfall events. On the other hand, in the second factor, ion dilution can be observed (i.e., the higher flow, the lower TDS values and lower concentrations of nitrogen and phosphorus compounds). Lenart-Boroń et al. (2016a) in their 2-year study indicated that the variability of nutrient concentrations and the abundance of bacteria in this sampling site is strictly associated with anthropogenic pressure in the form of inflow of either fresh or distant in time fecal contamination. The study conducted in the same site over a period of 3 years (Lenart-Boroń et al. 2016c) allowed to distinguish also the effect of seasonality—not only of anthropogenic pressure, which is typical of this area, but also of natural factors, such as increased surface runoff during heavy rainfall events. This site is situated in the close proximity to one of the largest ski resorts in the considered area and the effect of the development of ski infrastructure on the quality of water is evident in this site. Such problems of environmental effects are not restricted to the studied region but are observed in various parts of the world. Pickering et al. (2003) in their study on environmental effect of tourism, including ski infrastructure, on the protected areas of Australian Apls, indicated that inflow of untreated human waste downstream of the ski resorts is the most important factor. Also runoff from ski slopes is among the most important factors, followed by the deterioration of aquatic environment by extensive water intake for snowmaking. Microbiological contamination of water at the intake for the production of artificial snow of a ski resort may pose a potential health risks to the ski tourists. This is due to the fact that it has been observed that some portion of coliforms and *E. coli* may survive the process of snowmaking, resulting in the contaminated snow on the ski slopes (Lenart-Boroń et al. 2017a).

Finally, in Trybsz (No.4), factor 1 clearly indicates the effect of ion dilution during the periods of increased water flows, whereas in the second factor there is a positive correlation between microbiological indicators and flow rate, demonstrating leaching of bacteria from the soil cover into the river water. In contrast, in the study based on a shorter period of sampling, Lenart-Boroń et al. (2016a) classified this site as the one affected only by anthropogenic factors, such as the inflow of sewage. In the 3-year study, Lenart-Boroń et al. (2016b) observed the effect of both groups of factors—anthropogenic related to the municipal sewage inflow and natural associated with the seasonal changes, mostly in water temperature, which in turn affects the number of some bacterial groups. This site is situated downstream of the small town, Białka Tatrzańska, which is visited by huge numbers of tourists both during summer holidays and over the winter “high ski season,” i.e., from late December to early March. During winter holidays, there are often not enough places to accommodate all tourists visiting the area. All this results in a huge environmental impact of tourism and tourist-related infrastructure on the quality of water in this site. This includes both discharge of insufficiently treated sewage from the local treatment plant, inflow of untreated sewage from private guesthouses, and runoff from ski slopes or roads, as well as modification of the aquatic conditions by drawing of water for snowmaking in numerous ski resorts located not only in the Białka Tatrzańska town, but also upstream. Similar group of environmental impacts are observed in the Australian Alps (Pickering et al. 2003), in the boreal forest zone of Finland (Kangas et al. 2009) or South Korea (An and Breidenbach 2005).

The annual changes in physicochemical characteristics of the Białka river waters are clear and statistically significant. The occurrence of seasonal changes in the chemical composition of water highly depends on natural factors, such as hydrological and climatic conditions or biological activity of the catchment (Żelazny and Siwek 2012). In contrast, bacterial indicators of fecal contamination of water do not show statistically significant seasonal differences in the study area. Also Frenzel and Couvillion (2002) found, based on an example of 14 streams in Alaska, that there were no statistically significant differences in the concentration of bacteria. However, many studies demonstrated that the concentration of bacterial indicators shows seasonal variability (Edwards et al. 1997). In the case of the studied region, the prevalence of bacterial indicators of water quality changes throughout the year, but these changes result from the seasonal variation in the touristic activity in the area, which can vary from year to year. The number of bacteria in waters is affected by point and non-point sources of sewage inflow occurring in the study area (Lenart-Boroń et al. 2016a), and they are crucial in shaping the microbiological quality of water. The number of tourists visiting the area directly affects the river contamination, as it affects the amount of sewage produced and both—reaching the municipal treatment plant and illegally discharged directly into the river Białka (Lenart-Boroń et al. 2016a). The fact that the number of bacterial indicators in the selected sites of the river Białka is strongly affected by point sources of pollution, such as sewage discharge from the municipal treatment plant and illegal connections from private households, results in significant diurnal variations in the amount of pollutants reaching water of Białka (Lenart-Boroń et al. 2016b). This means that the time of the day when the samples are collected can significantly affect the obtained results, indicating that more thorough monitoring, i.e., based on more frequently collected samples, would produce more reliable results. Also—depending on the atmospheric conditions, the amount of sewage (both treated from the treatment plant and untreated from households) reaches the river, whose resources can be reduced due to the production of artificial snow to improve the conditions in the local ski stations (Krąż 2012b). The amount of water in the mountain river, such as Białka, can also change significantly during extreme weather conditions, such as floods. This is because of their high energy; mountain watercourses are highly vulnerable to environmental changes affecting their channels and catchments. Floods in mountain rivers are favored by the typically steep channel gradients and can be generated by various atmospheric events (Stoffel et al. 2016). Thus, in the studied area, it would be valuable to conduct detailed analyses of isolated events, such as flood waves.

Most studies show a positive correlation between the number of bacteria and flow rate values (Edwards et al. 1997; Sinclair et al. 2009). However, our studies indicate that this relationship is very weak. Nevertheless, it should be emphasized that the higher numbers of bacteria were recorded in waters during the high-water level than during low-water level periods. In contrast to microbiological indicators, physicochemical characteristics of water clearly depend on the flow rates. The higher the water flow, the lower EC, TDS, and NO_3_^−^ concentrations. During flood waves, river waters are diluted with low-mineralized precipitation waters or snowmelt waters. In the case of nitrogen compounds, their concentrations are clearly affected by the biological activity of the catchment. This is because during the growing period, nitrogen is assimilated by plants and the concentrations of its compounds are significantly lower, which is confirmed by other studies (Gardner and McGlynn 2009; Halliday et al. 2013). In turn, higher concentrations of nitrogen compounds are observed in autumn and winter, when the biological activity is definitely lower. On the other hand, the highest concentrations occur mostly in the initial snowmelt stage. Then the nitrogen compounds are leached out from the snow cover and soil (Johannessen and Henriksen 1978; Holko et al. 2006).

## Conclusions

This study showed that the considered region has a number of point and non-point sources of pollution, resulting from the specificity of the area, i.e., intensively developing tourism, especially winter tourism. This affects the dynamics of parameters influencing the quality of water. What remains constant is the microbiological pollution of water, which causes potential health risks to tourists, as water in the studied area is used for a wide range of recreational purposes.

It was shown that the development of ski infrastructure and—in more general terms—infrastructure related to mountain tourism negatively affects the quality of river water in the examined catchment. The impact of the point and non-point sources of pollution increases along the course of the river. The most downstream situated sampling site is subjected to a wide range of factors that deteriorate the water quality. The problems observed in the discussed catchment are common to other regions of the world, as the negative impact of mountain tourism, including ski infrastructure, is a common issue, reported in different countries.

Seasonal changes in the studied parameters were more clearly visible in the case of physicochemical parameters rather than for microbiological indicators of water quality. Along the studied river, the temporal changes in the number of bacteria are affected by point and non-point sources of pollution. In such cases, inflow of pollutants is also subject to temporal fluctuations, but their pattern is not that obvious as in the case of strictly seasonal changes.

Our study also showed the relationship between the water flow rate and the concentration of the analyzed factors. Again, this relationship is more evident in the case of physicochemical parameters than for microbial indicators. This demonstrates that the amount of bacteria in water is more associated with inflow of sewage (whose even small amounts may contain high concentrations of pollutants) rather than with non-point sources, such as surface runoff or snowmelt water. In order to more precisely identify the relation between concentrations of nutrients and flow rate, it would be worth conducting a detailed study of isolated events, such as flood waves.
